# The Effect of Luting Agent on the Fracture Resistance of Root Canal Treated Teeth Restored with Casting Post

**Published:** 2007-01-20

**Authors:** Sayed Shojaedin Shayegh, Kiumars Nazari Moghaddam, Maryam Khoroshy, Froogh Khoroshy

**Affiliations:** 1 *Department of Prostodontics, Dental School, Shahed University, Tehran, Iran*; 2 *Department of Endodontics, Dental School, Shahed University, Tehran, Iran*; 3 *Department of Operative Dentistry, Dental School, Isfahan University of Medical Sciences, Isfahan, Iran*; 4 *Department of Material Engineering, Isfahan University of Technology, Isfahan, Iran*

**Keywords:** Casting Post, Fracture Strength, Luting Cement

## Abstract

**INTRODUCTION:** The aim of this study was to determine the effect of different luting agents on fracture resistance of endodontically treated teeth restored with casting post.

**MATERIALS AND METHODS:** In this experimental study, forty extracted human maxillary central incisors teeth with the mean length of 23mm were randomly assigned in to four groups. All the studied teeth were caries free without any crack. After root canal treatment, the specimens were stored in 100% relative humidity at 37°C for 72h, and were decoronated 2mm above cementoenamel junction. The teeth in group1, 2, 3, and 4 received casting post and core and they were cemented with Zinc phosphate, Panavia F, Fuji Glass Ionomer, and Rely X, respectively. All teeth received 1.5 mm shoulder finishing line and 0.5mm bevel. Samples were then restored with complete coverage crowns and were loaded with an Instron universal testing machine. The cross-head speed was 0/02 cm/min and specimens were loaded with load values (Newton) computed at a speed of 1000 point/min, until the fracture happened. Loads were applied with 135 degree at middle lingual surfaces of the samples. Fracture loads were recorded. Data were analyzed by the one-way ANOVA test.

**RESULTS:** There was no significant difference between the fracture resistances of four test groups.

**CONCLUSION:** According to the results of this *in vitro* study, the type of luting cement had no influence on the fracture resistance of teeth**.**

## INTRODUCTION

The primary purpose of “post” is retention of “core” in a tooth with extensive loss of coronal tooth structure. However, space preparation for post causes certain risks for restorative procedures. Procedural accidents may occur during post-space preparation. Also, such teeth have been shown significantly shorter lifespan in comparison with vital teeth (1-2).

The substantially decreased structural integrity of the tooth because of the removal of tooth structure during endodontic access preparation, dowel-space preparation, and cavity preparation is generally an accepted reason for the increased failure rate (3-4).

Several classic studies have proposed that the dentin of root canal treated teeth substantially differs from the dentin of vital teeth (1-3). It was thought that the dentin in endodontically treated teeth was more brittle because of water loss and loss of collagen cross-linking (4).

Post and core are commonly advised to replace the lost coronal tooth structure and provide retention and resistance for the final restoration (5). It is generally accepted that bonded posts are more retentive than cemented posts and provide a better seal (6).

There are several types of cement available for permanent cementation of posts. The mechanisms which hold a post in a prepared canal contain: mechanical, micro mechanical, chemical, and in many cases a combination of these mechanisms are involved (7).

Cementation of posts has two main functions: retention and stress distribution, certainly the latter is more important due to transmitting functional stress from post to dentin. The luting could also be served as a modifier to forces, allowing better stress distribution (8).

Some studies showed that the use of low viscosity resin cements decreases the risk of root fracture (9). These cements have been proven to increase retention by bonding directly to not only the radicular dentin but also the post (10).

Numerous new luting cements and bonding adhesives with new mechanical and physical properties have been presented. The aim of the present *in vitro* study was to compare four luting cement on fracture resistance of the post and core restored teeth.

## MATERIALS AND METHODS

Forty intact maxillary central incisor teeth with similar dimensions, no fillings, no caries and no cracks at x2 magnification that might affect their fracture resistance to loading, were selected from a collection of extracted teeth and stored in a solution of neutral buffered formalin within one month.

Teeth with 10 mm coronal height and 13 mm root length were randomly assigned into 4 groups. Root length was measured from apex to the cementoenamel junction (CEJ) at buccal aspect. Buccolingual and Mesiodistal dimensions at the level of the cervical margin were also recorded with a digital caliper (Legtoon22, Switzerland). Root canals were prepared with K-files (Maillefer, Dentsply, Switzerland) via step-back technique. Apical preparation was completed with #35 K-file and the root canals were obturated by lateral condensation technique, using gutta-percha (Ariadent, Apadanatac, Iran) and zinc oxide eugenol root canal sealer (Roth international ltd., Chicago, USA).

The specimens were stored in 100% relative humidity at 37°C for 72h. The teeth were decoronated at 2mm above the CEJ with a diamond bur (Deatec, Switzerland) of high speed handpiece under continuous water coolant. All samples received chamfer finishing line preparations using a regular and fine tapered bur (Deatec, Switzerland) in a high speed hand piece including a 1-2 mm ferrule. Post rooms were prepared by removing filling material with Gates-Glidden, so that 5 mm of gutta-percha in the apical area was left.

Gates-Glidden #1-3 were used in order to enlarge the canals. Post-cores were made for each root using acrylic resin (Duralay, Reliance Dental, worth II) .The cores have similar length and tapering. The acrylic patterns were invested and casted with Ni-Cr alloy (Ramanium CS, Dentaurum, Pforzheim, Germany).

After casting and finishing, post-cores were cemented in following groups with:

Group A- Zinc phosphate cement (Detrey Division, Dentsply ltd., konstanz, Germany)

Group B- Fuji Glass Ionomer cement (GC International Corp., Tokyo, Japan)

Group C- Panavia F (kuraray, Osaka, Japan)

Group D- Rely X (Are.3M, ESPG, USA).

Impressions of the specimens were taken by using a polycyloxan impression material (President, Coltene, Switzerland) and full crowns were casted by using Ni-Cr Alloy (Ramanium CS, Dentauxum, Pforzheim, Germany). After casting and finishing, the crowns were cemented using zinc phosphate cement. The samples were subjected to thermocycling (6000 cycles at 5-55°C, dwell time 3, transfer time 5s) and were stored in 37°C sterile water for 15min, and were then embedded in cold curing resin up to 2mm beneath the CEJ.

The samples were then fixed into a metal hold in a universal testing machine (Zoeic1400 K. Germany), the loads were applied at 135 degrees angle to long axial, with a steel rod that has a rounded end. Loads were applied at an angle of 135 degrees in the middle of lingual surfaces of the teeth just 2 mm below the incisal edge.

The cross-head speed was 0/02 cm/min and specimens were loaded to fracture with load values (Newton) computed at a speed of 1000 point/min.

One-way analysis of variance (ANOVA) was used to compare the mean fracture load of each group.

## RESULTS

The means of force of fracture and standard deviations (SD) for four test groups are presented in Table 1. Statistical analysis of the data showed no significant difference between experimental groups.

## DISCUSSION

It is necessary to restore root canal treated teeth in order to preserve sound coronal tooth structure. No previous study has examined the effect of different luting cement on fractures resistance of teeth restored with casting post. The post's ability to anchor the core is critically important for successful reconstruction of the endodontically treated tooth. Loss of core retention results in crown removal. Retention of post in the root reflects the bonding process and the inherent strength of the post. A debonded or fractured post cannot retain the core and crown. So, it is important to choose suitable luting material for post-core.

In this study, selected teeth are approximately the same in length and diameter, so differences were not significant. Post, in present study, had 13 mm length which was similar to previous researches (11-13).

The load was applied at 135° the horizontal with a steel road which provides the most clinically comparable angle of loading in anterior teeth (14).

A variety of cross-head speeds have been applied by other researchers but this does not seem to be a crucial factor. There is a general consensus on its range from 0.5 (15) to 76.2 mm/min (16).

Four different types of luting cements were used in this study. Zinc phosphate cement as a non-adhesive luting agent during cementation has been extending into small irregularities of the dentine surface.

Glass Ionomer cements such as Fuji I have shown a moderately light bonding strength to enamel, dentine and Ni-Cr Alloys (7).

Panavia F which is based on micromechanical bonding was used in this study. Previous studies have showed that resin cements reduce the stress. Mendoza *et al.* found that luting resin cements were more resistant to fracture than zinc phosphate (17). Peroz *et al.* have recommended the use of adhesive cements for luting all types of posts because of more success rate. This study has demonstrated that different cements did not significantly alter the fracture resistance of single rooted teeth which restored by casting post (18).

**Table 1:** Fracture resistance of endodontically treated maxillary central incisor teeth after using four different luting cements for post core restorations in Newton.

**Groups**	**Mean**	**SD**
Zinc Phosphate cement	697.67	148.33
Fuji Glass Ionomer cement	758.35	119.3
Panavia F	711.2	112.5
Rely X	701.6	118.2

This finding was in conflict with Mendozs and Peroz *et al.* (17-18) who have reported favorable fracture resistance of teeth which cemented post-core with resin cements and unfavorable fracture of teeth post cemented by conventional cements.

## CONCLUSION

In spite of limitations of this *in vitro* study, there was no statistically significant difference between the fracture resistances of post-core restored teeth which were cemented by four different luting agents.
